# Flower and Pod Source Influence on Pea Weevil (*Bruchus pisorum*) Oviposition Capacity and Preference

**DOI:** 10.3389/fpls.2019.00491

**Published:** 2019-04-24

**Authors:** Thaïs Aznar-Fernández, Diego Rubiales

**Affiliations:** Institute for Sustainable Agriculture, Consejo Superior de Investigaciones Científicas, Córdoba, Spain

**Keywords:** antixenosis, *Bruchus pisorum*, deterrence, oviposition, *Pisum sativum*, preference

## Abstract

*Bruchus pisorum* is an insect pest causing major damage to pea seeds worldwide. Control is difficult and limited resistance is available. In this work we studied the effects of pollen and pod source on insect fecundity and oviposition by comparing resistant and susceptible *Pisum* spp. accessions and non-host (*Lathyrus sativus* and *Vicia faba*) species. A first no-choice assay revealed that the source of flower offered to adults for feeding might retard oviposition (the case of *V. faba*), reduce fertility (*Pisum sativum* ssp. *syriacum*, *P. fulvum*, and *V. faba*) or increase adult mortality (*V. faba* and *P. sativum* ssp. *syriacum*). A second no-choice assay with all adults fed with pollen of the same pea cultivar showed significant effect of the source of pods offered. Oviposition was reduced on pods of some resistant *Pisum* accessions, but particularly low on pods of the non-hosts, being retarded if ever happening and coupled with high mortality of adults. This was confirmed in a third experiment consisting on dual-choice assays showing reduced egg laying in *V. faba*, *L. sativus*, *P. fulvum*, and *P. sativum* ssp. *syriacum* compared to the commercial variety pea used as a control (Messire).

## Introduction

Field pea (*Pisum sativum* L.) is the first temperate grain legume produced in Europe and the second in the world (FAOSTAT, 2016). Their use extends to dry peas for animal fodder and green peas for human consumption. In addition, as a legume, it brings environmental benefits (Rubiales and Mikic, 2014).

Pea is constrained by a number of pests and diseases with the pea weevil (*Bruchus pisorum* L., Coleoptera: Bruchidae, *Bp*) being a serious concern worldwide. *Bp* causes yield losses of up to 50% (Clement et al., 2002; Keneni et al., 2011). After a period of hibernation, *Bp* females feed on pollen and oviposit on pods. Once the egg has hatched, the emerging larvae penetrate through the pods into the seeds, where they feed on the endosperm (Teshome et al., 2015). This reduces seed yield and devaluates seed quality and marketability (Brindley and Hinman, 1937; Nikolova and Georgieva, 2015). Effective chemical control requires repeated treatments at flowering and fruiting stages (Michael et al., 1990; Horne and Bailey, 1991) coupled with post-harvest fumigations in order to prevent adult apparition inside storehouse (Clement et al., 2009). Biological control (Huis et al., 1990) and management by intercropping (Teshome et al., 2016) have been attempted with no definitive results. Use of resistant cultivars offer a suitable alternative but they are not available so far, although some genetic resistance had been reported in pea germplasms (Teshome et al., 2015; Aznar-Fernández et al., 2017). Availability of unattractive or repellent genotypes for oviposition would help in designing crop mixtures to manage the pest (Shelton and Badenes-Perez, 2006; Ratnadass et al., 2012) and in breeding resistant cultivars.

The objective of this work was to identify host and non-host plant genotypic effects on sexual maturity of *Bp* adults by studying the pre-oviposition period and oviposition capacity (Wäckers et al., 2007), as well as the oviposition preference and *Bp* longevity in order to disclose the resistant mechanism present in some resistant accessions to *Bp*.

## Materials and Methods

### Field Screenings

Thirteen *Pisum* spp. accessions showing different levels of *Bp* infestation were selected from a previous study (Aznar-Fernández et al., 2017). In order to corroborate data, pea accessions were field screened during 2014/15 season at Córdoba (Latitude 37°51′25″N; Longitude 04°48′10″W; Altitude 117 m) and at Escacena (Latitude 37°22′01″N; Longitude 06°32′29″W; Altitude 192 m), Spain (Table 1). The experimental design consisted of a complete block design with three randomized repetitions. Each accession was represented by 25 seeds planted in a 50 cm long row, with a separation of 50 cm between accessions. Córdoba’s trial was drip irrigated whereas Escacena’s trial was rain fed. No pesticides or herbicides were applied and only mechanical weeding was done when needed. When natural *Bp* infestation was first observed in the area, *Bp* adults were released on the plots at the rate of 3–4 adults/m^2^. These had been collected from cv. Messire seeds infested during the previous season and stored at 4°C.

**Table 1:** Pea weevil seed infestation (%SI) on 13 *Pisum* accessions under field conditions (2014–2015).

					%SI ± SEM	
Accession	Synonym^†^	Origin	Species	Subspecies	Córdoba	Escacena
P26	PI 116056	India	*P. sativum*	*sativum*	44.7 ± 6.4	40.3 ± 0.3
P36	PI 343988	Turkey	*P. sativum*	*sativum*	34.6 ± 1.4	29.7 ± 2.7
P37	PI 505080	Cyprus	*P. sativum*	*sativum*	49.2 ± 1.2	27.7 ± 8.2
P38	PI 505092	Cyprus	*P. sativum*	*sativum*	42.9 ± 7	23.6 ± 4
P39	PI 505111	Syria	*P. sativum*	*sativum*	38.8 ± 5.4	18.8 ± 9.8
P624	IFPI 2348	Ethiopia	*P. sativum*	*arvense*	48.1 ± 1.7	22.7 ± 1.8
P638	IFPI 2362	Ethiopia	*P. sativum*	*arvense*	43.3 ± 1.8	30.2 ± 1.2
P639	IFPI 2363	Ethiopia	*P. sativum*	*arvense*	47.3 ± 7.2	32.7 ± 12.9
P646	IFPI 2370	Ethiopia	*P. sativum*	*arvense*	40.6 ± 2.7	41.0 ± 5.3
P656	IFPI 3250	Syria	*P. fulvum*		22.5 ± 3.8	22.5 ± 3.8
P665	IFPI 3280	Syria	*P. sativum*	*syriacum*	5.6 ± 3.1	2.1 ± 0.4
P669	IFPI 3330	Turkey	*P. sativum*	*elatius*	16.0 ± 3.8	7.0 ± 1.2
Messire		France	*P. sativum*	*sativum*	81.7 ± 3.5	45.2 ± 3.6
			Location Mean ± SE:		39.6 ± 3.0	26.41 ± 2.3

*^†^PI-numbers: accessions provided by USDA, United States; IFPI-numbers: accessions provided by ICARDA, Syria. SEM, standard error of the mean.*

At maturity, seeds were manually harvested, threshed and assessed for seed infestation (SI) by opening 100 seeds of each repetition through the cotyledons (Aznar-Fernández et al., 2017).

### Bioassays Under Controlled Conditions

#### General Conditions

Following field data (Table 1), pea cv. Messire was our susceptible control; the genotypes to be evaluated were: P669 (*P. sativum* ssp. *elatius*), P665 (*P. sativum* ssp. *syriacum*), and P656 (*P. fulvum*), showing moderate resistance in field. The non-hosts, faba bean (*V. faba* cv. Brocal) and grasspea (*L. sativus* cv. Titana) were selected from other studies (data not shown).

*Bp* adults used in all experiments came from infested seeds of pea cv. Messire, that were collected from the trials described above and stored in paper envelopes at 4°C. Adults of *Bp* emerging from these seeds were sexed by the presence (male) or absence (female) of a small spine on the tibia of the middle leg (Yus Ramos, 1976). Thereafter, *Bp* were separated into falcon tubes and were stored again at 4°C (Mendesil et al., 2016). Forty-eight hours before the experiment, the *Bp* adults were recovered from the fridge and place under chamber conditions (27°C) with water provided; those showing greater movement were selected for the experiments.

In addition, host and non-host plant species selected to develop the assays, were grown in the field under a mesh protected shelter. To ensure a sufficient supply of clean flowers and pods at the required stage, seeds were sown at various planting dates. Plants were drip irrigated and no chemicals were applied on the plots or surroundings.

Assays were performed in a growth chamber under optimal conditions for *Bp* (27 ± 2°C, 16L: 8D, 70% RH). Experiments were conducted inside cylindrical plastic cages (12 cm diameter, 10 cm depth) with a hole on the wall (12 cm^2^) covered with an anti-trips patch in order to facilitate transpiration as well as for avoid possible leaks or external intrusions. Wrinkled napkin paper was placed at the bottom of the cages to provide nooks where the weevils could hide. Tap water was provided inside an Eppendorf sealed with cotton and hooked on the wall of the cages. Flowers and pods used in each assay were placed in Eppendorf’s with tap water and sealed by parafilm (Supplementary Figure 1).

#### Flower Source Effect on *Bp* Oviposition in No-Choice Assay

The experimental design consisted on 10 random replications per accession, each one consisting in a cage with five flowers of the test accession plus two pods in late flat and early swollen stage of pea cv. Messire. Four *Bp* females and two males were released per cage and allowed to feed, mate, and oviposit (Clement et al., 2002) (Supplementary Figure 1A). Flowers and pods were provided and replaced on alternate days. Cages were monitored daily to assess the days till the first oviposition and the number of eggs laid that day. To have a general estimate of *Bp* mortality, according to the number of dead weevils inside cages, a symbol value was given as follows: −/+) all cages with less than 4 dead adults; ++) about half of the cages with 5–6 dead adults; and +++) most of the cages with 5–6 dead adults (Table 2).

**Table 2:** Effect of flower genotype intake and pod offered on *Bruchus pisorum* oviposition in no-choice assays (see Figure 1).

		Flower source effect^†^			Pod source effect^‡^		
		Number of eggs laid on pods	Days till first oviposition	*Bp* mortality	Number of eggs laid on pods	Days till first oviposition	*Bp* mortality
					
Accession	Species	(Average ± SE)		(Average ± SE)	(Average ± SE)	(Average ± SE)	
Messire	*P.s*. ssp. *sativum*	25.9 ± 4.6	5.5 ± 0.2	−/+	25.0 ± 4.2	5.0 ± 0.29	−/+
P669	*P.s.* ssp. *elatius*	19.7 ± 0.8	5.5 ± 0.2	−/+	23.4 ± 0.2	4.5 ± 0.25	−/+
P656	*P. fulvum*	14.3 ± 3.1	5.8 ± 0.5	−/+	14.0 ± 3.2	5.6 ± 0.56	−/+
P665	*P.s*. ssp. *syriacum*	18.0 ± 1.8	5.5 ± 0.2	++	10.1 ± 3.2	5.8 ± 0.53	−/+
Titana	*Lathyrus sativus*	21.1 ± 2.8	5.6 ± 0.3	−/+	1.2 ± 0.2^T^	13.4 ± 0.47^T^	++
Brocal	*Vicia faba*	20.0 ± 2.3^α^	8.0 ± 0.4^α^	+++	−^§^	−^§^	+++

*^†^Effect of host and non-host flowers on Bp oviposition capacity on pea cv. Messire. ^‡^Effect of host and non-host pods on oviposition of Bp feed with flowers of pea cv. Messire. ^T^Only six cages showed oviposition; ^α^only four repetitions showed oviposition; ^§^ Some eggs were observed but never on pods; SE, standard error.*

#### Pod Source Effect on *Bp* Oviposition on Pods in No-Choice Assay

The experimental design consisted of 15 random replicates per accession formed by a cage as described above, where 4 females and 2 males of *Bp* were freed. Each repetition consisted on two pods of the accession to test (Table 2) in the late flat and early swollen pod stages to allow *Bp* oviposition. To feed and stimulate the oviposition, five flowers of the control Messire were also included per cage. Flowers and pods were provided and replaced on alternate days. P669 accession was used when there was still no presence of neoplasm formation (Np). In order to assess pod genotype effect on *Bp* oviposition, cages were monitored daily to assess the days till the first oviposition and the number of eggs laid this day. *Bp* mortality was also estimated as indicated above (−/+, ++, +++).

#### Evaluation of *Bp* Oviposition Preference in Dual Choice Assay

The bioassay consisted on cages as described above, containing tap water and two pods, one of cv. Messire and the other from the accession to test. The pods offered for oviposition were at late flat and early swollen stages and distributed on opposite sides of the cage (Supplementary Figure 1B). Two sexually mature females, previously fed on cv. Messire flowers, and two males were released to allow the *Bp* oviposition as described above. Ten repetitions per combination were performed. To avoid possible stresses, four fresh flowers of the control pea cv. Messire were provided. The number of eggs laid over each pod was assessed 24 h after the infestation (hai).

### Statistics

Data of field screenings (% of SI) was submitted to an analysis of variance (ANOVA) with accession and locality as fixed factors. For no choice bioassays, data of count variables was analyzed with a generalized linear model (GLM) run with Poisson error distribution. Preference for oviposition was analyzed using the Student’s *t*-test. Analyses were made by using Statistix 10^®^(Analytical Software, Tallahassee, FL, United States).

## Results

### Field Screenings

Results showed higher infestation levels at Córdoba than at Escacena (Table 1). ANOVA for %SI showed significant genotype and environment effect (*P* = 0.0001), meanwhile G × E interaction was not (Table 3); interestingly both locations showed similar weather conditions except for accumulated rain (Table 4). However, accessions P665, P669, and P656 showed the lowest %SI in both environments evaluated. Thus, these three accessions were selected to perform bioassays under controlled conditions with cv. Messire as control, which showed the highest %SI values in both environments.

**Table 3:** Analysis of variance for *Bruchus pisorum* seed infestation percentage (%SI) of the 13 pea genotypes in the two environments evaluated (Córdoba and Escacena).

Source	DF	SS	MS	*F*	*P*
Environment (E)	1	2950.2	2950.18	42.56	0.0001
Genotype (G)	12	12618.6	1051.55	15.17	0.0001
E × G	12	1535.6	127.96	1.85	0.0645

*DF, degrees of freedom; MS, mean square; G × S, term of genotype × season interaction.*

**Table 4:** Environmental description of the trials of the study.

Environment	Season	Av. Temp (°C)	Av. Humidity (%)	Accu. Rainfall (mm)	Accu. Rad. (W/m^2^)
Escacena	2014–2015	15.2	66.1	130.0	17.7
Córdoba		15.0	66.8	164.1	17.4

*The parameters (Av. Temp., average temperature; Av. Humidity, average humidity; Accu. rainfall, accumulated rainfall; Accu. Rad., accumulated radiation) are given for the crop season (from sowing till harvest date).*

### *Bruchus pisorum* Bioassays Under Controlled Conditions

#### Effect of Flower Source on *Bp* Oviposition in No-Choice Assay

Significant differences were found among tested accessions for the number of eggs laid (*df* = 58; *P* = 0.0001) although not for the number of days till first oviposition (*P* > 0.05). Females fed on P665 and P656 flowers, laid significantly fewer eggs (Table 2). *Bp* fed on faba bean cv. Brocal showed retarded oviposition, although large proportion of the adults fed on Brocal died. Mortality was moderate on adults fed on P665, but low on those fed on the remaining accessions.

#### Effect of Pod Source on *Bp* Oviposition in No-Choice Assay

The number of eggs laid and the number of days till first oviposition showed significant differences among tested accessions (*df* = 62; *P* = 0.0001). The number of eggs laid on pods was high on cv. Messire (25 eggs/pod), being similar in P669 (*circa* 23) but significantly reduced on pods of P656 and P665 (14 and 10 eggs/pod, respectively) and nil or almost negligible for the non-hosts *V. faba* cv. Brocal and *L. sativus* cv. Titana. Number of days required for first oviposition was similar among *Pisum* accessions (range 4.5–5.8 days) and highly retarded on pods of *L. sativus* cv. Titana (13.4 days). No eggs were laid on pods of *V. faba* but on any place in those cages, either on the parafilm or the cage walls (Supplementary Figure 2). Only in one repetition 1 egg was observed on a *V. faba* pod, which was not included in the analyses. In addition, large proportion of the adults offered pods of *V. faba* died. Mortality was higher on *L. sativus* pods cages, in comparison with those fed on the remaining accessions.

In order to corroborate the high mortality observed in cages with *V. faba* pods, six additional repetitions were performed under the same conditions described on the Section “Pod Source Effect on Bp Oviposition on Pods in No-Choice Assay.” In all cages *Bp* died before the oviposition (data not shown).

#### Evaluation of *Bp* Oviposition Preference in Dual Choice Assay

**FIGURE 1:**
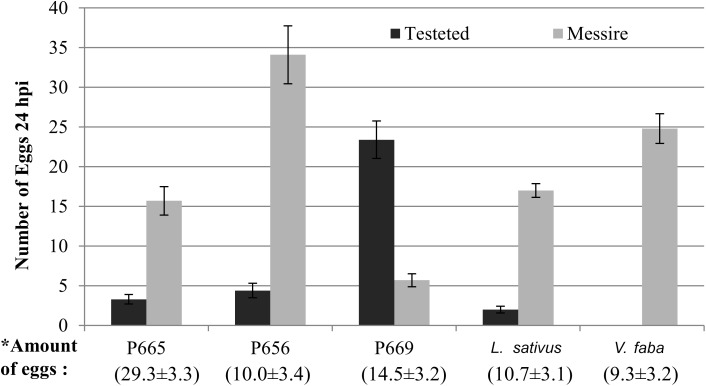
Oviposition of *B. pisorum* in dual choice assays under controlled conditions. Columns show the distribution of eggs laid over pods 24 h after infestation. Bars showed the mean ± standard error (SE). In brackets is the mean of total amount of eggs laid per combination (Messire + tested genotype) ± standard error (SE).

In dual choice assays Messire was generally preferred for oviposition. Accessions confronted with Messire showed significantly reduced oviposition in cages containing P665, *L. sativus* or P656 (Table 5). No eggs were laid on *V. faba* pods. Conversely, P669 was preferred for oviposition than Messire (Table 5 and Figure 1). In addition, the total amount of eggs laid on P656 – Messire combination was significantly higher than for the remaining combinations (Figure 1).

**Table 5:** *Bruchus pisorum* oviposition preference in Dual Choice assay between five different genotypes and Messire (positive control).

Species	Genotype	*T*	*P*-value
*Pisum*	P669	−3.91	0.003
*Pisum*	P656	3.81	0.004
*Pisum*	P665	3.73	0.005
*Lathyrus*	Titana	9.54	0.0001
*Vicia faba*	Brocal	5.65	0.0003
	*df* = 9		

*T-test results showed preference for oviposition on Messire at higher T levels.*

## Discussion

Resistance to pea weevil is a major priority for pea breeding. Field screenings corroborated the environmental effect on seed infestation and highlighted the higher resistance of P656, P665, P669 accessions in both environments evaluated in agreement with Aznar-Fernández et al. (2017). Bioassays reported here corroborate the influence of both flower and the pod on *Bp* oviposition. The importance of pollen and nectar consumption in *Bp* oviposition is largely known (Clement, 1992; Wäckers et al., 2007). In our study, the source of pollen influenced the number of eggs laid. In addition, *V. faba* caused a high *Bp* mortality and retarded oviposition, thereby suggesting that pollen and nectar probably affect the sexual maturity of *Bp* females (Pesho and Van Hounten, 1982). However, females fed on non-host species flowers, such as *L. sativus*, are sexually mature which is in agreement with observations of Barry and O’Keeffe (1984) who reported that sexual maturation of *Bp* females depends on the amounts of pollen ingested rather than differences in nutritional quality of different pollens. Moreover, this behavior could prolong *Bp* life-span (Pajni, 1981) and benefit their dispersal by providing a source of energy to sustain flight after hibernation (Clement, 1992). Our study shows that the sources of flowers offered might reduce (the case of various *Pisum* accessions) or even retard oviposition and cause adult mortality (the case of *V. faba*). This could be due to the primary metabolites, which are important feeding stimulants for Coleoptera, and might be different between species and genotypes (Wäckers et al., 2005). Further studies are needed to discern if the retarded and reduced oviposition of females fed on *V. faba* flowers are due just to amount of pollen and/or nectar eaten or to anti-nutritional effects. The mortality of adults fed with *V. faba* and P665 flowers might suggest some anti nutritional effect (Table 2). Interestingly, accessions P665, P656, P669, and Brocal showed flower pigmentation known to be associated with condensed tannins (Wang et al., 1998). Tannins are widely recognized as plant defense compounds against herbivore insects (Barbehenn and Constabel, 2011) and could act as deterrents for feeding under natural conditions.

On the other hand, there was a strong effect of pod offered on oviposition preference on the number of eggs laid, and days till first oviposition on non-host species. This suggests the crucial role of plant genotype on weevil oviposition. Oviposition was particularly affected on *V. faba* and *L. sativus* with a marked reduction in the number of eggs laid and a delayed start of oviposition on *L. sativus*. There was also a significant reduction of number of eggs laid on *P. sativum* ssp. *syriacum* (P665) and *P. fulvum* (P656). As described above, P665 accession showed purple pigmentation also in pods. Antixenosis and antibiosis on *P. fulvum* pods has been previously described (Hardie and Clement, 2001; Clement et al., 2002). *Bp* oviposition repellence or deterrence on pods might be due to structural defense mechanisms such as the touch, thickness, color, presence of trichomes (Edwards and Singh, 2006; Mendesil et al., 2016) wax layer (Chang et al., 2006) and also due to secondary metabolites such as volatiles (Bruce et al., 2011; Ceballos et al., 2015) or plant defense responses to *Bp* presence (Bennett and Wallsgrove, 1994). The length of pea pods could also interfere in *Bp* preference for oviposition (Hardie and Clement, 2001); however, this would not interfere in our trials since in the late flat and early swollen stage of pods from our bioassays displayed similar lengths.

Our results show strong deterrence against non-host *V. faba* cv. Brocal, forcing females to oviposit elsewhere but not over *V. faba* pods (Supplementary Figure 2). In addition, mortality displayed inside cages with non-host species suggests that both (*V. faba* and *L. sativus*) influence on *Bp* lifespan.

*Pisum sativum* ssp. *elatius* accession P669 showed consistent reduced seed infestation in field screenings under multiple environments [see section “Field Screenings,” in agreement with Aznar-Fernández et al. (2017)]. This might be due to neoplasm (*Np*) formation often observed in this accession, although the effect has been not quantified. Neoplasm formation has been reported to reduce the efficiency of *Bp* larval penetration (Doss et al., 1995), being the reduction in oviposition associated with the level of neoplasm formation (Mendesil et al., 2016). Our experiments showed that young pods of P669 are not deterrent, suggesting that reduction of infestation under field conditions might indeed be due to effects of neoplasm. However, we used young pods, before neoplasms were formed, and therefore cannot discern whether the reduced infestation of P669 is due to neoplasm reducing oviposition and/or hampering successful larval occlusion and penetration on pods.

In dual-choice and no-choice assays, accessions P665, P656 and *L. sativus* showed lower preference for oviposition than Messire. However, in dual-choice assays, when P656 and Messire were studied together in the same experimental cage, the number of eggs was the highest, suggesting no interference of P656 on *Bp* egg lying. In addition, no eggs were laid on pods of *V. faba* on dual and no-choice assays; this egg-lying deterrence deserves further investigations. As described before, several traits such could play a major role on *Bp* oviposition preference. It has also been described in non-host plants that several metabolites and pheromones might act as oviposition-deterrence on non-target insects (Cook et al., 2007). Results of this study suggest the use of P665 and *V. faba* as promising combinations in intercropping (Finch and Collier, 2012); being both push-pull strategies which modify the pest behavior in order to reduce *Bp* pressure on the crop (Cook et al., 2007). Another interesting finding of this work is the suitability of *L. sativus* pollen for *Bp* oviposition, but not over *Lathyrus* pods. This discourages the pea-Lathyrus mixed-cropping, since it would increase the oviposition pressure over pea pods. Field studies on the effect of mixed-cropping as suggests above, need to be conducted in order to arrive at definitive conclusion.

## Author Contributions

TA-F designed and developed the assays and wrote the manuscript. DR supervised the study and contributed to the data interpretation and writing of the manuscript.

## Conflict of Interest Statement

The authors declare that the research was conducted in the absence of any commercial or financial relationships that could be construed as a potential conflict of interest.
