# Supplementary material for: Flower and Pod Source Influence on Pea Weevil (Bruchus pisorum) Oviposition Capacity and Preference
Source: Front Plant Sci. 2019 Apr 24;10:491. doi: 10.3389/fpls.2019.00491 (PMC6491779; doi:10.3389/fpls.2019.00491)

Supplemental file 1. Plastic cages used in bioassays to evaluate the effect of flower and pod genotype on *B.pisorum* oviposition. (A) No choice assays to study the effect of host and non-host flowers on *Bp* oviposition capacity on pods of pea cv. Messire; (B) Dual choice assay to study the preference of *Bp* oviposition over host and non-host pods.

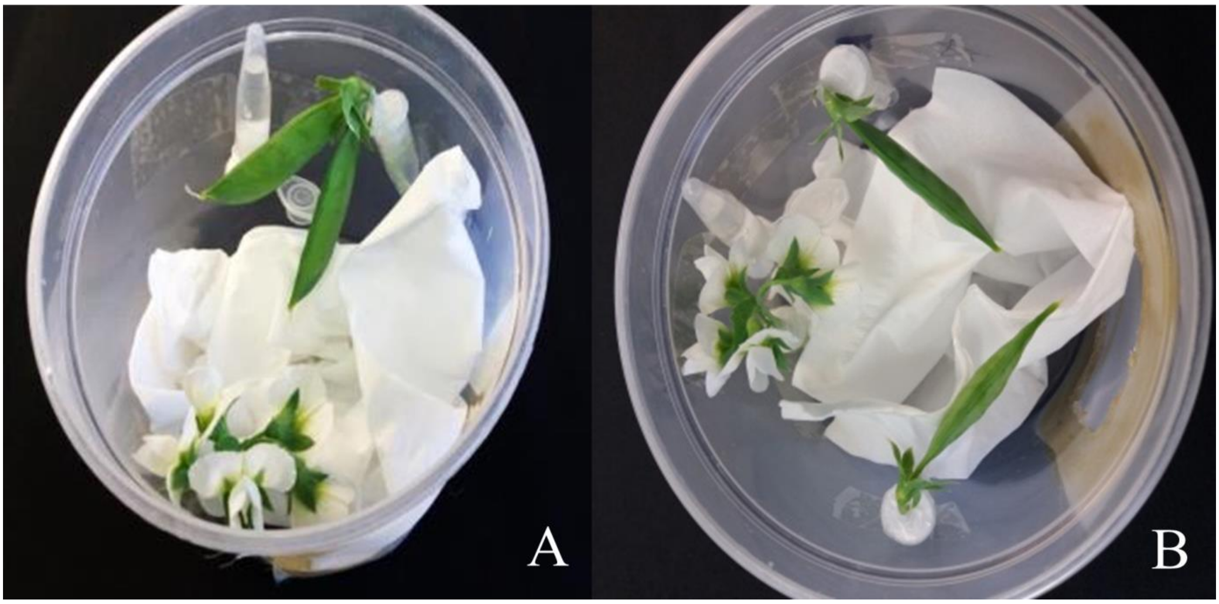

Supplement: Supplementary file 1 [file Image_1.pdf]
