# Supplementary material for: Flower and Pod Source Influence on Pea Weevil (Bruchus pisorum) Oviposition Capacity and Preference
Source: Front Plant Sci. 2019 Apr 24;10:491. doi: 10.3389/fpls.2019.00491 (PMC6491779; doi:10.3389/fpls.2019.00491)

Supplemental file 2. Effect of pod genotype on oviposition of *B. pisorum* females previously feed with pollen of cv. Messire. (A) Pea cv. Messire pod with abundant eggs laid; (B) *Faba bean* cv. Brocal pod without eggs laid; (C) and (D) details of (B) showing eggs lied elsewhere (on top of the parafilm and ppendorf tube) but not on pod.

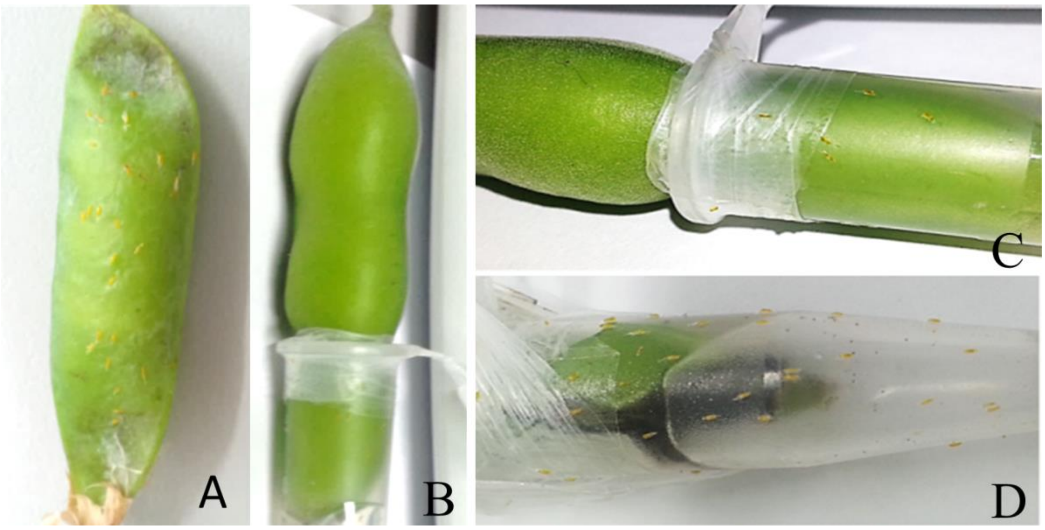

Supplement: Supplementary file 2 [file Image_2.pdf]
